# Yolk @ cage-Shell Hollow Mesoporous Monodispersion Nanospheres of Amorphous Calcium Phosphate for Drug Delivery with High Loading Capacity

**DOI:** 10.1186/s11671-017-2051-7

**Published:** 2017-04-13

**Authors:** Suping Huang, Chunxia Li, Qi Xiao

**Affiliations:** 1 State Key Lab of Powder Metallurgy, Central South University, Changsha, 410083 Hunan China; 2 School of Resources Processing and Bioengineering, Central South University, Changsha, 410083 Hunan China

**Keywords:** Amorphous calcium phosphate, Yolk @ cage-shell hollow nanospheres, DOX · HCl, Loading capacity

## Abstract

In this paper, yolk-shell hollow nanospheres of amorphous calcium phosphate (ACP) are prepared, and its loading capacity is investigated by comparing with that of solid-shell hollow structure ACP and cage-shell hollow structure ACP. Results show that the products are yolk @ cage-shell of ACP with large shell’s pores size (15-40 nm) and large cavity volume. Adsorption results show that the loading capacity of yolk @ cage-shell hollow spherical ACP is very high, which is more than twice that of hollow ACP and 1.5 times of cage-like ACP. The main reasons are that the big shell’s pore size contributes the large molecular doxorubicin hydrochloride (DOX · HCl) to enter the inner of hollow spheres easier, and the yolk-shell structure provides larger interior space and more adsorption sites for loading drugs.

## Background

Over the past decades, many efforts have been devoted to design novel controlled drug-delivery systems, which are superior to commercial administrated drugs in terms of dosage, due to their high delivery efficiency [1], low side effects [2], and low toxicity [3]. To date, various polymer [4], inorganic [5], and inorganic/organic hybrid materials [6] with diverse structures and shapes have been employed as vehicles for drug delivery. Particularly, calcium phosphate salts have gained considerable attention in the delivery of different drugs due to their excellent biocompatibility, low toxicity, excellent nonimmunogenicity and osteoconductive properties [7–10]. However, the relatively low surface area and small pore volume may limit their application. Thus, developing a kind of functional hollow calcium phosphate spheres should be highly potential, not only due to their biomedical characteristics but for their large interior space and tunable porous shell, which is suitable for loading more drugs and diffusing the drug molecules through the channels freely.

In order to enhance the loading capacity, various calcium phosphate materials with diverse morphologies and size have been prepared [11–14], such as calcium phosphate composite nanoparticles [15, 16], hydroxyapatite hollow microspheres [17–19], hydroxyapatite microtubes [20, 21], hydroxyapatite assembled hollow fibers [22], hydroxyapatite nanowires [23], and flower-like hierarchically nanostructured hydroxyapatite hollow spheres [24]. Among the different morphological nanostructures, yolk-shell hollow spheres with porosity [25] are more advantageous for applications in biomedical fields such as loading drug, protein or DNA molecules, due to their different specific surface areas and morphologies [26, 27]. However, the reports about yolk-shell calcium phosphate particles are very little, and the particles reported previously could not meet the loading requires because their big yolk size compared with the shell, which results in the smaller interior space and low loading capacity [25].

On the other hand, the shell’s pore sizes of hollow sphere nanoparticles are very important for the delivery of drugs into cells. Large molecular/volume drugs are difficult to enter the small shell’s pores, and mostly adsorb on the surface of hollow spheres. It is a challenge to synthesize yolk-shell hollow structures of ACP with small particle sizes but simultaneously bigger pore sizes and larger interior space for the delivery of large molecular weight therapeutics.

In this paper, we will prepare a kind of yolk-shell hollow mesoporous nanospheres of calcium phosphate with bigger pore sizes and large interior space, and compare the loading capacity of yolk-shell structure with the solid-shell hollow structure and cage-like hollow structure. At the same time, the effect of yolk-shell structure’s pore sizes and cavity volume on the loading capacity will be investigated.

## Methods

### Materials

All chemicals used throughout the experiments were of analytical grade and without further purification. Calcium nitrate [Ca(NO_3_)_2_ · 4H_2_O, 99 wt%] as a source of Ca was purchased from Tianjin Hengxing Chemical Co., Ltd., China. Phosphorus pentoxide (P_2_O_5_, 98 wt%) as a source of P was purchased from Tianjin Kermel Chemical Reagent Co., Ltd., China, and an ammonia solution (NH_3·_H_2_O, 25–28 wt%) was purchased from Zhuzhou Quartzification Glass Co., Ltd., China. Anhydrous ethanol (CH_3_CH_2_OH, 99.7 wt%) was purchased from Tianjin Zhiyuan Chemical Reagent Co., Ltd., China.

### Synthesis of Phenol-Formaldehyde Resin Spheres (PRs)

Monodisperse phenol-formaldehyde resin spheres (PRs) were synthesized by using resorcinol and formaldehyde solution as precursors. Generally, ammonia aqueous solution (NH_4_OH, 25 wt%, 0.1~0.3 mL) was mixed with a solution containing absolute ethanol (EtOH, 0~28 mL) and deionized water (H_2_O, 0~28 mL) (with totally amount of 28 mL) to prepare PRs with different sizes. After stirring for more than 1 h, different amounts of formaldehyde solution and resorcinol were added to each of the reaction solutions and stirred at 30 °C for 24 h, and subsequently heated at 100 °C for 24 h under a static condition in a Teflon-lined autoclave. The solid product was recovered by centrifugation and air-dried at 100 °C for 48 h [28].

### Synthesis of Amorphous Calcium Phosphate Nanospheres (ACPs)

Ca(NO_3_)_2_ · 4H_2_O (0.059 g), P_2_O_5_ (0.011 g), and PRs (0.100 g) were dissolved in three portions of anhydrous ethanol for 50 mL, respectively. Then, the solution containing P_2_O_5_ was added into the PRs solution. After 30 min of ultrasonic treatment, the Ca(NO_3_)_2_ solution was dropped into the mixture. Meanwhile, ammonia solution (NH_3_ · H_2_O, 0.001 mol L^−1^, 10 mL) was added dropwise into the mixture and reacted for 24 or 48 h. The whole process of the reaction was carried out under magnetic stirring. The precipitate was collected and washed alternately with anhydrous ethanol for 3 times by centrifugation (8000 rpm, 5 min), followed by drying at 50 °C for at least 24 h. Finally, the dried powder was calcinated up to 500 °C under air atmosphere with heating rate 2 °C/min and 10 °C/min (named ACP-24-2, ACP-48-2 and ACP-48-10, respectively, the number denotes the process parameter). In the process of calcination, the temperature was to keep heat-preservation at 100, 250, 500 °C for 1, 1, and 4 h respectively.

### Drug Loading

DOX · HCI was dissolved in deionized water to a concentration of 10 mg mL^−1^. Twenty milligrams of ACP nanoparticles was dispersed in 10 mL of the DOX solution. The mixture was stirred at room temperature for 24 h. Then, the DOX · HCI concentration of the supernatant was measured by UV-visible spectrophotometry at 480 nm. Then, the loading capacity was calculated by the equation as follows:

$$ Q = \left({C}_0- C\right)* V/ m $$

In the equation, *Q* (in mg g^−1^) is the amount of DOX · HCl adsorbed; *C*_0_ and *C* (in mg mL^−1^) are the concentrations of the solution containing of DOX before and after adsorption, respectively; *V* (in mL) is the volume of the solution; and *m* (in g) is the amount of ACPs.

### Characterization

The phase of powders was analyzed by an X-ray diffraction (XRD, Rigaku D/max-2550) with a monochromatic Cu K_α_ radiation (*λ* = 1.5419 Å) using a voltage of 40 kV and a current of 250 mA. The data was recorded by a step size of 0.02° s^−1^ and a scanning range from 2θ = 10 to 80°. The Fourier transform infrared spectroscopy (FT-IR, Nicolet 6700) analysis was used to identify the chemical and structural compositions in ACP particles, which were detected by mixing with KBr powder with the scanning range from 4000 to 400 cm^−1^. The morphology and size of ACP were observed by using a field-emission scanning electron microscope (FESEM, Quanta FEG 250), and a JEM-2100 F transmission electron microscope (TEM) at an acceleration voltage of 200 kV. The nitrogen adsorption-desorption isotherms were obtained on a Quantachrome Autosorb automatic analyzer to determine the specific surface area and pore volume of the ACP hollow nanospheres. The amount of DOX · HCl adsorbed on the ACP hollow nanospheres was measured using UV-visible absorption spectrophotometer at 480 nm.

## Results and Discussion

**Fig. 1:**
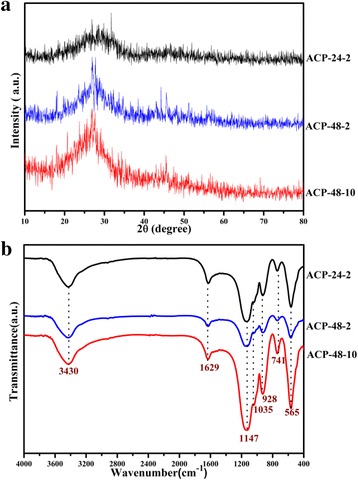
XRD patterns (**a**) and FT-IR spectra (**b**) of the ACP nanospheres obtained under different conditions

Figure 1a, b shows the XRD patterns and FT-IR spectra of the PR and ACP nanospheres, respectively. All XRD patterns and FT-IR spectrum are similar to each other, separately. As Fig. 1a shows, there are no obvious diffraction peaks of the crystalline calcium phosphate, and the broad and curve bread peak at about 2θ = 27° indicates the amorphous phase of synthesized particles [29]. In FT-IR spectra (shown in Fig. 1b), the broad absorption peak 3430 cm^−1^ and 1629 cm^−1^ is ascribed to the –OH group of water molecules [30, 31]. The absorption peaks at 1147, 1035, and 928 cm^−1^ are the P-O stretching vibration bands of P-O, and 565 cm^−1^ is bending vibration band of P-O, which is characteristic bands of PO_4_^3−^ ions. The single peak at 565 cm^−1^ illustrates that the products are amorphous calcium phosphate, while the anisotropic local electric field of crystalline apatite partially splits into an apparent doublet absorption band between 500 and 600 cm^−1^ [32]; and the intense absorption peak located at around 1147 cm^−1^ is attributed to the characteristic of ACP molecules [33], which both are consistent of the XRD results.

**Fig. 2:**
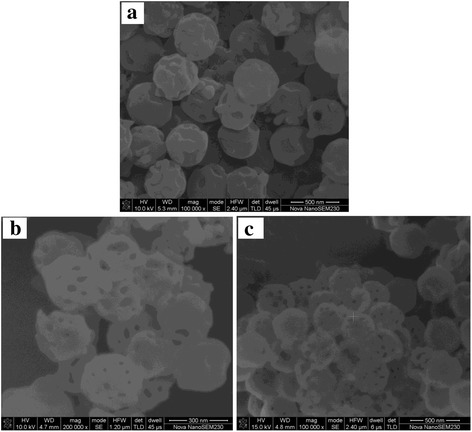
SEM images of ACP-24-2 (**a**), ACP-48-2 (**b**), and ACP-48-10 (**c**)

Figure 2 displays the SEM images of samples. It shows that the samples of ACP after calcination are all hollow spherical particles, but the shell’s morphologies are different. The product ACP-24-2 is solid-shell hollow spherical ACP. and only one or two big pores, formed due to gas emission during PS pyrogenic decomposition, can be seen on the outer shell. The products of ACP-48-2 and ACP-48-10 are also hollow spheres, but there were many big pores on the shell. And, the shell’s pore size is between 15 and 40 nm. The average sizes of as-synthesized products ACP-24-2 and ACP-48-2 are around 400 nm, while the particles size of ACP-48-10 is about 300 nm, which indicates that the average size of samples decreases with the increasing of heating rate.

**Fig. 3:**
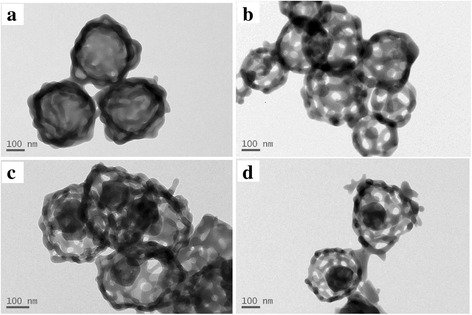
TEM images of the products. **a** ACP-24-2. **b** ACP-48-2. **c, d** ACP-48-10

Figure 3 shows the TEM images of samples. It can be seen that the product ACP-24-2 (Fig. 3a) is a spherical hollow particle, and there are only one or two big pores on the shell, which may be produced due to the gas discharging during the process of pyrolysis. The product ACP-48-2 (Fig. 3b) is a single-layer hollow spherical ACP without yolk. And, there are many big pores on the shell, which look like spherical cages, so it is named as cage-like hollow ACP. The product ACP-48-10 (Fig. 3c, d) is yolk-shell hollow spherical ACP. On the shell, there are many big pores, looks like a cage sphere, and the yolk is a solid sphere with diameter 120 nm. So, product ACP-48-10 is named as yolk @ cage-shell hollow ACP spheres.

**Fig. 4:**
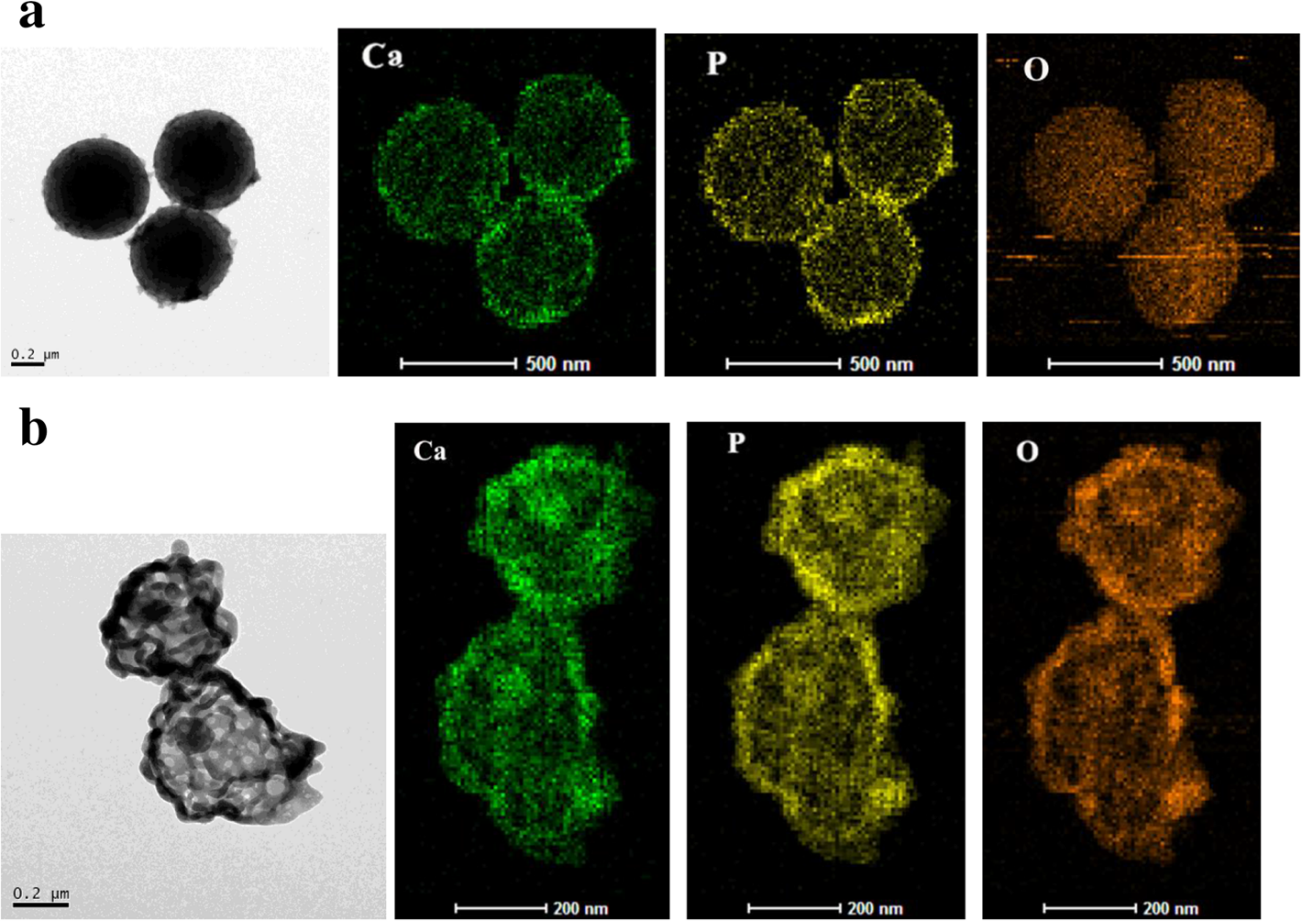
The element mapping of yolk @ cage-shell hollow ACP sphere before calcination (**a**) and after calcination (**b**)

In order to further investigate the Ca, P, and O distribution within the as-synthesized yolk @ cage-shell hollow ACP before and after calcination, the element mapping of Ca, P, and O were performed in Fig. 4. It can be seen that, before calcination, the O was homogeneously distributed within the PR sphere, and the P and Ca were mainly distributed on the ACP coating; a few P and Ca entered into the PR sphere; and after calcination, P, O, and Ca were homogenously distributed on the cage shell and the yolk, which indicated that the cage shell and the yolk are amorphous ACP.

**Fig. 5:**
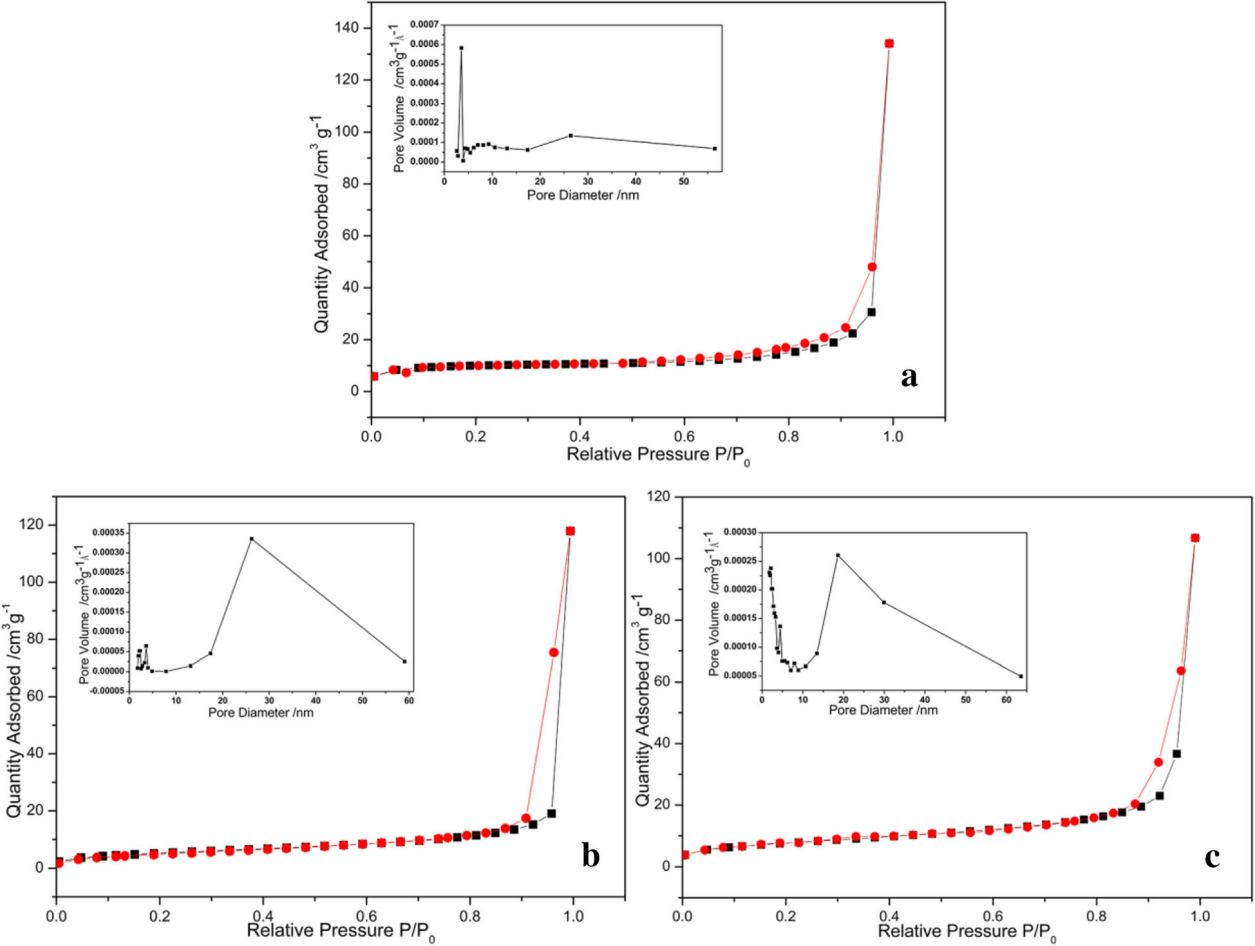
Nitrogen adsorption-desorption isotherms and pore size distribution graphs of ACP-24-2 (**a**), ACP-48-2 (**b**), and ACP-48-10 (**c**)

Figure 5 shows the nitrogen adsorption-desorption isotherms of as-synthesized ACP nanoparticles. All curves are similar and exhibit the type IV isotherms with type H_3_ hysteresis loops according to Brunauer-Deming-Deming-Teller (BDDT) classification [34], illustrating the presence of mesoporous structures (2–50 nm). In our study, the hysteresis loops can be attributed to the hollow cores and mesopores [35, 36]. The plot of pore size distribution is calculated on the basis of Barrett-Joyner-Halenda (BJH) method from the desorption branch of the isotherm. The plot of pore size distribution of ACP-24-2 shows a narrow pore size distribution ranging from 2–4 nm with a distinct peak, which indicates that the shell of ACP-24-2 is a mesoporous hollow sphere with small pores (2–4 nm), which is in accordance with the SEM and TEM results. The plot of pore size distribution of other two samples displays a broad size distribution ranging from 15 to 40 nm. These pore sizes correspond closely with the pore size on the outer shell observed in TEM micrographs, suggesting that these pores on the outer shell are responsible for the pore size distribution. Namely, the outer shells of the other two samples are cage-like spheres with big pores (14–40 nm).The parameters of samples synthesized are shown in Table 1. By comparison, the product ACP-48-10 has the largest surface area (31.7668 m^2^ g^−1^), pore volume (0.2008 cm^3^ g^−1^), and average pore size (33.5726 nm). The characterization of large pore size, yolk-shell structure may play a decisive role in drug adsorption.

**Table 1:** The surface area (S_BET_), pore volume, average pore size, and adsorption for samples synthesized under the different conditions

Sample	S_BET_^a^ /m^2^ g^−1^	Pore volume^b^ /cm^3^ g^−1^	Adsorption amount /mg g^−1^
ACP-24-2	17.7495	0.0613	350.4
ACP-48-2	18.9721	0.1808	490.7
ACP-48-10	31.7668	0.2008	1181.9

^a^Calculated from Barrett-Emmett-Teller (BET) equation

^b^Calculated using Barrett-Joyner-Halenda (BJH)

To demonstrate the potential application of yolk @ cage-shell hollow spherical ACP as delivery carrier/vehicles, doxorubicin hydrochloride (DOX · HCl), an anticancer drug, is chosen as a model drug. The DOX · HCl loading capacities of these three ACP particles with different morphologies are evaluated at pH 7.4 in deionized water. The adsorption amount is calculated by the depletion of DOX · HCl in the solution measured by UV-visible spectrophotometer at 480 nm, as shown in Table 1. The adsorption amounts of ACP-24-2, ACP-48-2, and ACP-48-10 are 350.4, 490.7, and 1181.9 mg g^−1^_,_ respectively. The loading capacity of yolk @ cage-shell hollow spherical ACP is twice more than that of ACP-24-2 and ACP-48-2, which indicates that yolk @ cage-shell hollow spherical structure contributed to the drug’s loading.

**Fig. 6:**
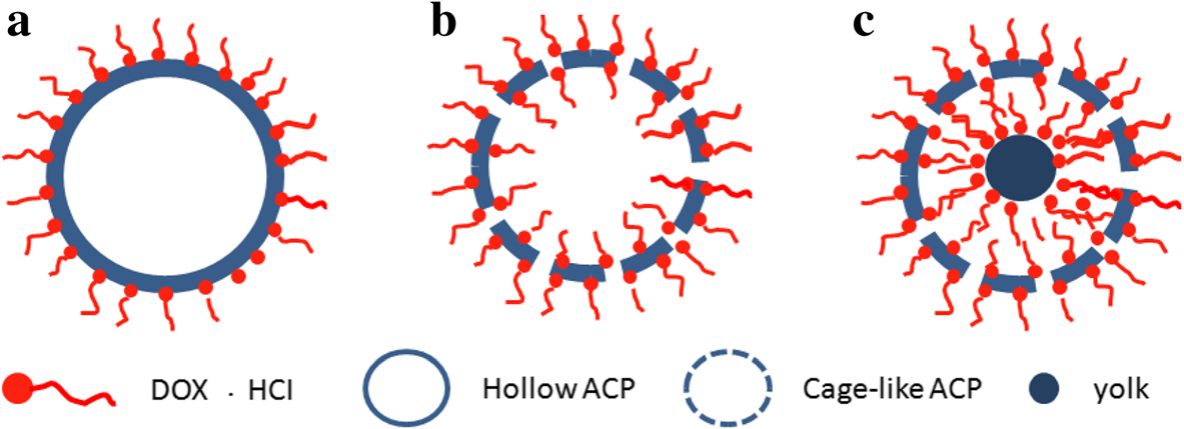
The adsorption behaviors of DOX · HCl on the ACP particles with different morphologies. **a** hollow spheres without big pores on shell **b** cage-shell hollw spheres **c** yolk @ cage-shell hollow spheres

The differences in DOX · HCl adsorption behaviors may be explained by different morphologies and specific surface areas. The particle size and cavity volume of ACP-24-2 and ACP-48-2 are similar, but the loading capacity of ACP-48-2 is higher than that of ACP-24-2, which indicates that the pore sizes play an important role in adsorption. DOX · HCl is large molecular (cube with dimensions 1.07 × 4.51 × 1.44 nm). It is very difficult for DOX · HCl to transfer these pores which diameter is smaller than 5 nm. The pore sizes of ACP-24-2 are less than 5 nm (as shown in Fig. 5a), so DOX · HCl cannot transfer the shell and mainly adsorbed on the outer surface of shell (as shown in Fig. 6a). The pore sizes of ACP-48-2 ranged from 14–50 nm, DOX · HCl can easily enter into the inner of the ACP spheres and can adsorb on the inner and outer surface of shell (as shown in Fig. 6b). ACP-48-10 is yolk @ cage-shell hollow structure nanospheres with big pore sizes (14–50 nm) and larger interior space (yolk diameter less than 100 nm), which are suitable for DOX · HCl entering through the channels freely and adsorbing on the outer and inner of cage shell, and surface of yolk (as shown in Fig. 6c). On the other hand, the difference in DOX · HCl adsorption behavior between the ACP-24-2, ACP-48-2, and ACP-48-10 may be depended on the different specific surface area. By comparing ACP-24-2, ACP-48-2, and ACP-48-10, it is observable that the trend of DOX · HCl adsorption amount is similar to that of specific surface area, which indicates that specific surface areas may also play a crucial role in loading capacity.

Compared with other types of core-shell nanospheres reported previously [11–13, 15], the yolk @ cage-shell hollow ACP nanospheres have smaller particle size, bigger shell’s pore size and larger cavity space, which are suitable for more DOX · HCl entering through the channels freely and adsorbing on the yolk surface and shell surface (outer and inner surface). They are the reasons why the loading capacity of the yolk @ cage-shell hollow ACP nanospheres is larger than that of others. On the other hand, the Zeta potentials of DOX · HCl and the yolk @ cage-shell hollow ACP nanospheres in deionized water at pH 7.4 are 12.1 and −19.2 mV, respectively. The strong attractive electrostatic force between ACP nanospheres and DOX · HCl enhances the loading capacity.

## Conclusions

Yolk @ cage-shell hollow mesoporous monodispersion nanospheres of amorphous calcium phosphate with big pores (from 15 to 40 nm) and large interior space are prepared. The loading capacity of yolk @ cage-shell hollow spherical ACP is 1181.9 mg g^−1^_,_ which is more than twice that of solid-shell hollow spheres 350.4 mg g^−1^ and cage-like hollow spheres (490.7 mg g^−1^). The reasons are that the big pore sizes make large DOX · HCl moleculars to enter the hollow spheres easily, the yolk-shell structures with the biggest specific surface area and interior space provide more adsorbing sites for DOX · HCl moleculars, which greatly enhanced the loading capacity.

## Abbreviations

ACP: Amorphous calcium phosphate
DOX•HCl: Doxorubicin hydrochloride
FESEM: Field-emission scanning electron microscope
FT-IR: Fourier transform infrared spectroscopy
PRs: Phenol-formaldehyde resin spheres
TEM: Transmission electron microscope
XRD: X-ray differaction
